# A Multilevel Bayesian Approach to Improve Effect Size Estimation in Regression Modeling of Metabolomics Data Utilizing Imputation with Uncertainty

**DOI:** 10.3390/metabo10080319

**Published:** 2020-08-06

**Authors:** Christopher E. Gillies, Theodore S. Jennaro, Michael A. Puskarich, Ruchi Sharma, Kevin R. Ward, Xudong Fan, Alan E. Jones, Kathleen A. Stringer

**Affiliations:** 1 Department of Emergency Medicine, University of Michigan, Ann Arbor, MI 48109, USA; keward@med.umich.edu; 2 Michigan Center for Integrative Research in Critical Care (MCIRCC), University of Michigan, Ann Arbor, MI 48109, USA; xsfan@umich.edu; 3 Michigan Institute for Data Science (MIDAS), Office of Research, University of Michigan, Ann Arbor, MI 48109, USA; 4 Department of Clinical Pharmacy, College of Pharmacy, University of Michigan, Ann Arbor, MI 48109, USA; tjennaro@med.umich.edu; 5 Department of Emergency Medicine, University of Minnesota, Minneapolis, MN 55455, USA; mike-em@umn.edu; 6 Department of Biomedical Engineering, University of Michigan, Ann Arbor, MI 48109, USA; rusharma@umich.edu; 7 Department of Emergency Medicine, University of Mississippi Medical Center, Jackson, MS 39216, USA; aejones@umc.edu; 8 The NMR Metabolomics Laboratory, Department of Clinical Pharmacy, College of Pharmacy, University of Michigan, Ann Arbor, MI 48109, USA; 9 Division of Pulmonary and Critical Care Medicine, Department of Internal Medicine, School of Medicine, University of Michigan, Ann Arbor, MI 48109, USA

**Keywords:** hierarchical modeling, nuclear magnetic resonance spectroscopy, Bayesian statistics, missing values, imputation, multiple test corrections

## Abstract

To ensure scientific reproducibility of metabolomics data, alternative statistical methods are needed. A paradigm shift away from the *p*-value toward an embracement of uncertainty and interval estimation of a metabolite’s true effect size may lead to improved study design and greater reproducibility. Multilevel Bayesian models are one approach that offer the added opportunity of incorporating imputed value uncertainty when missing data are present. We designed simulations of metabolomics data to compare multilevel Bayesian models to standard logistic regression with corrections for multiple hypothesis testing. Our simulations altered the sample size and the fraction of significant metabolites truly different between two outcome groups. We then introduced missingness to further assess model performance. Across simulations, the multilevel Bayesian approach more accurately estimated the effect size of metabolites that were significantly different between groups. Bayesian models also had greater power and mitigated the false discovery rate. In the presence of increased missing data, Bayesian models were able to accurately impute the *true* concentration and incorporating the uncertainty of these estimates improved overall prediction. In summary, our simulations demonstrate that a multilevel Bayesian approach accurately quantifies the estimated effect size of metabolite predictors in regression modeling, particularly in the presence of missing data.

## 1. Introduction

Challenges of scientific reproducibility have become increasingly prevalent due to selective reporting, *p*-hacking, pressure to publish, and low statistical power [1,2]. Surveyed researchers report a ‘better understanding of statistics’ as key to combatting the reproducibility crisis, and others have been revolting against the use of the *p*-value as an indicator of significance [1,3]. Specifically, these scientists argue against a null-hypothesis testing framework and that categorization of results as either ‘statistically significant’ or ‘non-significant’ based on a *p*-value often leads researchers to incorrect conclusions [2]. ‘Statistically significant’ results also tend to be upwardly biased in magnitude, causing replication or validation studies to be underpowered and fail a null-hypothesis testing framework [4]. Moreover, Gelman et al. have suggested that a focus on magnitude (Type M) and sign (Type S) errors of estimated effect sizes may be more useful in regard to ensuring scientific reproducibility [5,6]. Avoiding these aforementioned errors and producing robust effect size estimates that are of the same sign (i.e., positive or negative) and close in magnitude to the true effect size, are critical for the replication of a scientific finding. To fight the problems associated with reliable replication, the social sciences have embraced the idea of the ‘New Statistics’ that emphasizes uncertainty and interval estimation over frequentist null-hypothesis testing [7,8].

While the field of metabolomics has made important strides to ensure reproducibility, the approaches have often focused on data generation and analytical reproducibility over new statistical approaches [9,10,11,12]. As such, null-hypothesis testing remains prevalent, and scientific reproducibility remains a challenge in metabolomics, in part, because of the need for multiple testing of up to several thousands of metabolites. A common approach in the analysis of metabolomics studies is univariate regression modeling of metabolites associated with a specified outcome [13]. A typical approach to address the testing of multiple hypotheses involves correcting the *p*-value for the number of statistical tests conducted, which limits the Type I error rate or the risk for false positives. Common methods include the Bonferroni correction or the Benjamini–Hochberg (B–H) correction of a false discovery rate (FDR) [14]. Importantly, since these approaches focus on the *p*-value, they do not address upward bias of the magnitude of significant results or mitigate Type M and Type S errors. A shift away from null-hypothesis testing toward the embracement of effect size estimation with uncertainty for the statistical analysis of metabolomics data may result in enhanced reproducibility and more informed study designs.

Widespread adoption of multilevel Bayesian statistics applied to metabolomics data represents an opportunity to take the ideals of ‘New Statistics’ and apply a methodological framework in line with how science has always been done—using prior knowledge to inform the generation of new evidence in a way that iteratively improves our estimation of the truth. A primary advantage of Bayesian methodology is that it allows for the incorporation of prior knowledge with the observed data to make better predictions. Bayesian methodology, with its emphasis on incorporation of priors with the observed data, results in cumulative knowledge improvement while also emphasizing precision of a model’s estimates [15]. Multilevel modeling is a technique that improves effect size estimation by performing partial pooling and shrinking point estimates toward each other. This inherently deflates the risk for Type I error upon multiple testing and significantly reduces Type M and Type S errors in effect size estimation [16]. Another benefit of multilevel Bayesian modeling is that it can be easily extended to model challenges inherent to metabolomics data, for example the presence of missing data. A common form of missing data in metabolomics is the occurrence of left-censored missing data, which is typically caused by measurements made below the limit of detection of the assay or other specific analytical platform limitations [13]. Common imputation strategies, such as assigning a minimum value, can lead to biased effect size estimation. Bayesian methods have been shown to improve imputation of missing values and can account for uncertainty in the imputations [17,18]. Importantly, the inherent uncertainty of those imputed values, which is typically ignored, may be accounted for in regression analysis. This may provide further overall improvement in effect size estimation.

In our work reported here, our overarching aim was to test the ability of a Bayesian approach to improve effect size estimation in metabolomics data compared with other multiple testing correction approaches. To achieve our aim, we: (1) determined if a multilevel Bayesian modeling strategy can adjust for multiple testing for metabolomics data by controlling power and FDR; (2) assessed if a multilevel model improves the efficiency in the estimation of effect sizes for univariate variable ranking situations; and (3) tested whether ‘soft’ imputation further improves effect size estimation in the presence of missing data. We designed a simulation to model metabolomics data and assess the effectiveness of different multiple testing correction approaches. We then implemented two multilevel Bayesian logistic regression models. One model operated on a dataset that had no missing or imputed values. The second model followed a novel two-stage imputation design. In stage one, our model estimated the mean and variance of missing values using a multivariate Bayesian regression model within a left-censoring framework. Subsequently, in stage two, we fitted our multilevel logistic regression model using the estimated mean and variances of the missing values. We call this ‘soft’ imputation as compared to ‘hard’ imputation, since we fit a distribution to each missing value instead of a single value. We compared our multilevel models against standard logistic regression with appropriate methods for correction of multiple testing in a simulation of nuclear magnetic resonance (NMR) metabolomics data. Finally, we fit our two-stage Bayesian model using two existing metabolomics datasets: one generated by NMR [19,20,21] and the other by gas chromatography (GC) mass spectrometry (MS) [22], in order to assess model performance and determine the practical limitations of the work.

## 2. Results

### 2.1. A Multilevel Bayesian Approach Has Higher Power, Controls for False Discovery, and More Reliably Estimates Metabolite Effect Size across a Variety of Simulated Scenarios

**Figure 1:**
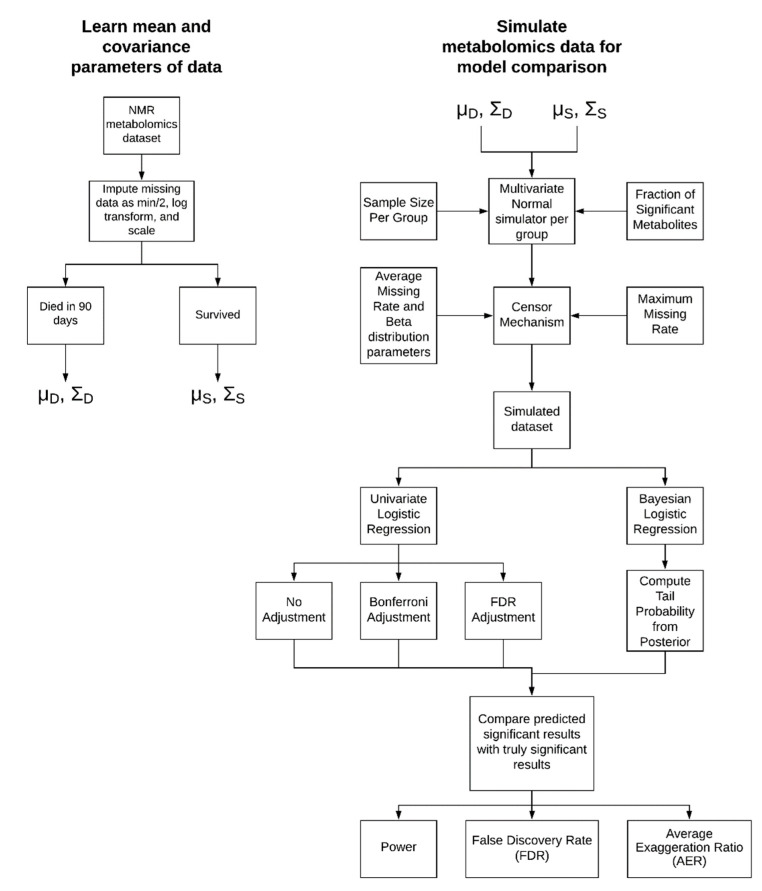
Simulation and model workflow. We began with a metabolomics dataset from patients with septic shock and prepared the data according to standard methods. We learned the mean and covariance of two outcome groups: survivors and non-survivors. Various parameters were adjusted to generate a simulated dataset corresponding to unique experimental conditions. We then ran a Bayesian logistic regression model and standard logistic regression +/− corrections for multiple testing to compare predicted results to the true results of the simulation.

Our simulations are based on ^1^H-NMR metabolomics data from two outcome groups of patients with septic shock (survivors and non-survivors). For both groups, we generated data from a multivariate normal distribution with the same means and covariance as the original data. We altered the sample size per group and the fraction of metabolites that are ‘truly’ different based on patient mortality. Two hundred-simulations for unique combinations of sample size per group and fraction of truly associated metabolites were completed (Figure 1).

**Figure 2:**
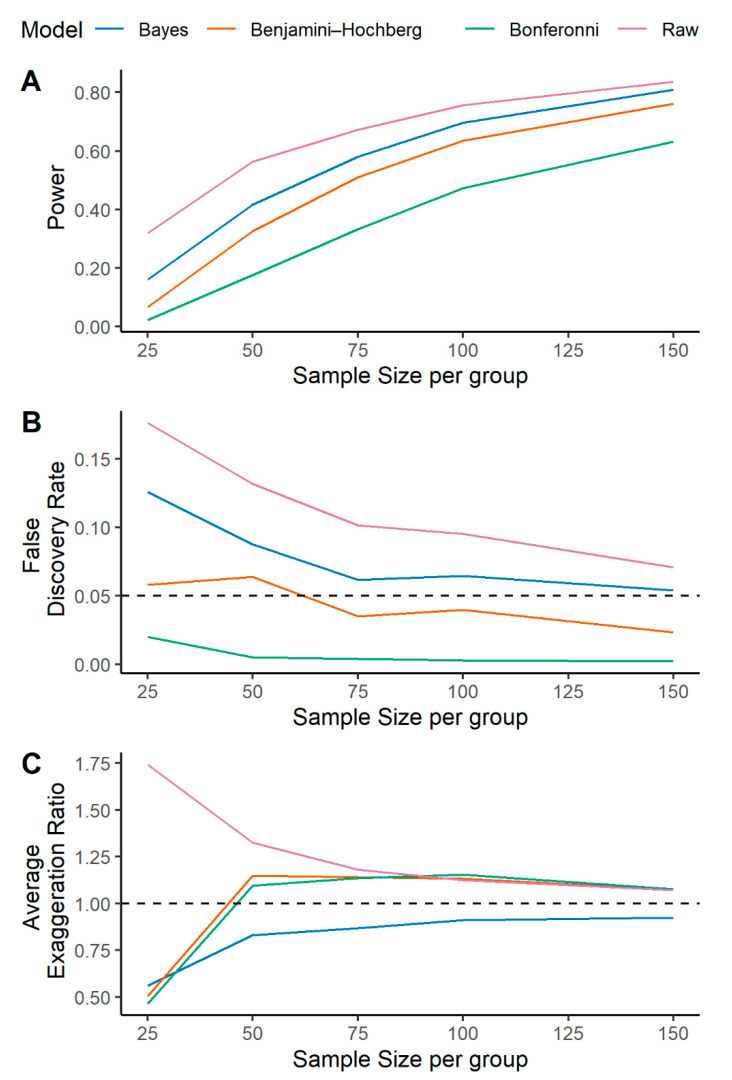
A multilevel Bayesian approach offers increased power and controls for false discovery while providing a more accurate estimation of metabolite effect size relative to other statistical correction approaches. A multilevel Bayesian model (blue lines) and a standard logistic regression (labeled raw, purple lines) were fit on a simulated metabolic dataset where 40% of metabolites were defined to be significantly different between groups (survivors vs. non-survivors). Logistic regression models were further adjusted for multiple testing according to Bonferroni (green lines) and Benjamini–Hochberg (orange lines). Models were fit at different sample sizes per group without the presence of missing data as described in the methods. Model predictions are provided as: (**A**) Power or True positive rate (TPR); (**B**) False Discovery Rate (FDR); (**C**) Average exaggeration ratio (AER) in estimated effect size. This is defined as the mean error over the set of metabolites that were significant and true (*ST*) for each model.

The univariate association of individual metabolites and sepsis mortality in the simulated data were conducted by standard logistic regression, logistic regression with a Bonferroni correction, logistic regression with a B-H FDR correction, and by a multilevel Bayesian approach (Figure 1). The power, FDR, and the average exaggeration ratio (AER) in estimated effect sizes for each model are presented as a function of sample size in Figure 2. Here, the exaggeration ratio for a given metabolite is a proxy for Type M error and is defined as the absolute value of the estimated effect size divided by the true parameter value [5]. The AER is simply the mean value across true positive metabolites.

The multilevel Bayesian model provides greater power for detecting significant metabolites relative to the Bonferroni or B-H FDR correction (Figure 2A). While the Bonferroni correction limits FDR to the greatest degree, the Bayesian model limits the FDR relative to an unadjusted logistic regression (Figure 2B). Importantly, for metabolite compounds simulated to be associated with mortality, the AER is considerably lower in the Bayesian model (Figure 2C). Similar patterns for power and FDR were observed when the fraction of significant metabolites was increased to 0.7 (Supplementary Figure S1 panels A and B), but the Bayesian approach offered superior effect size estimation with an AER of approximately 1 across sample size (Supplementary Figure S1 panel C). As such, the multilevel Bayesian approach more reliably estimates a metabolite’s effect size and limits the risk for Type M error.

Model performance as a function of the fraction of significant metabolites (holding sample size constant) is shown in Supplementary Figure S2. When a smaller fraction of metabolites is different between survivors and non-survivors, the Bayesian approach offers less control over FDR (Supplementary Figure S2 Panel B). The average effect size estimates for standard logistic regression methods are inflated (i.e., AER > 1) across fractions of significant metabolites, while the Bayes model offers conservative estimates when the fraction is low and steadily approaches an AER ~ 1 as the fraction increases (Supplementary Figure S2 Panel C). The model performance for all simulations across sample sizes and fraction of significant metabolites are provided in Supplementary Table S1.

### 2.2. Imputation Incorporating Uncertainty Improves Predicted Metabolite Concentration

**Figure 3:**
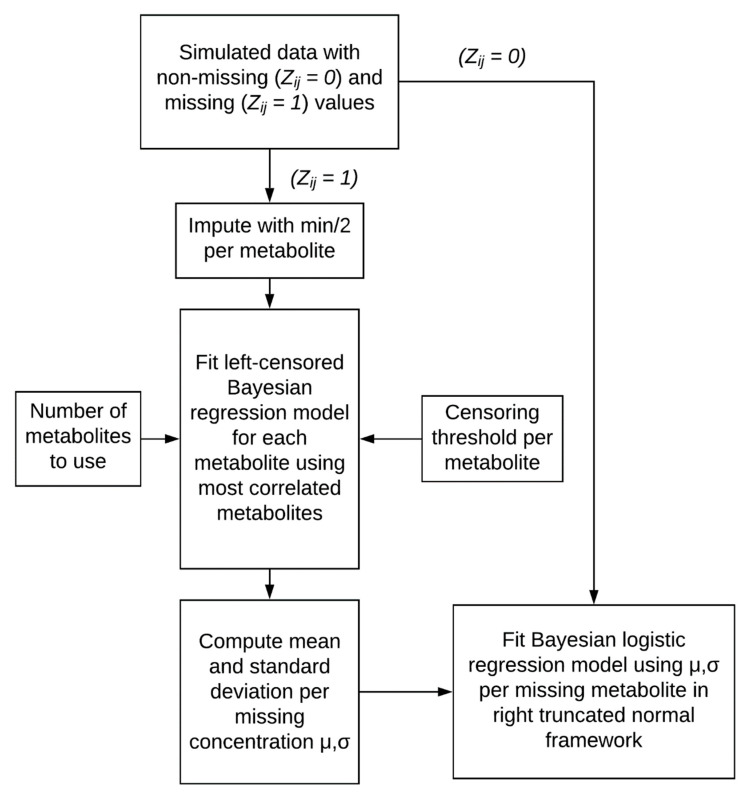
Two-stage ‘soft’ imputation methodology. We used Bayesian linear regression to impute missing metabolite observations, based on a censoring threshold and a user-defined number of correlated metabolites. The uncertainty in the imputed value, approximated by the standard deviation of the missing concentration, is accounted for upon fitting the subsequent multilevel Bayesian logistic regression.

**Figure 4:**
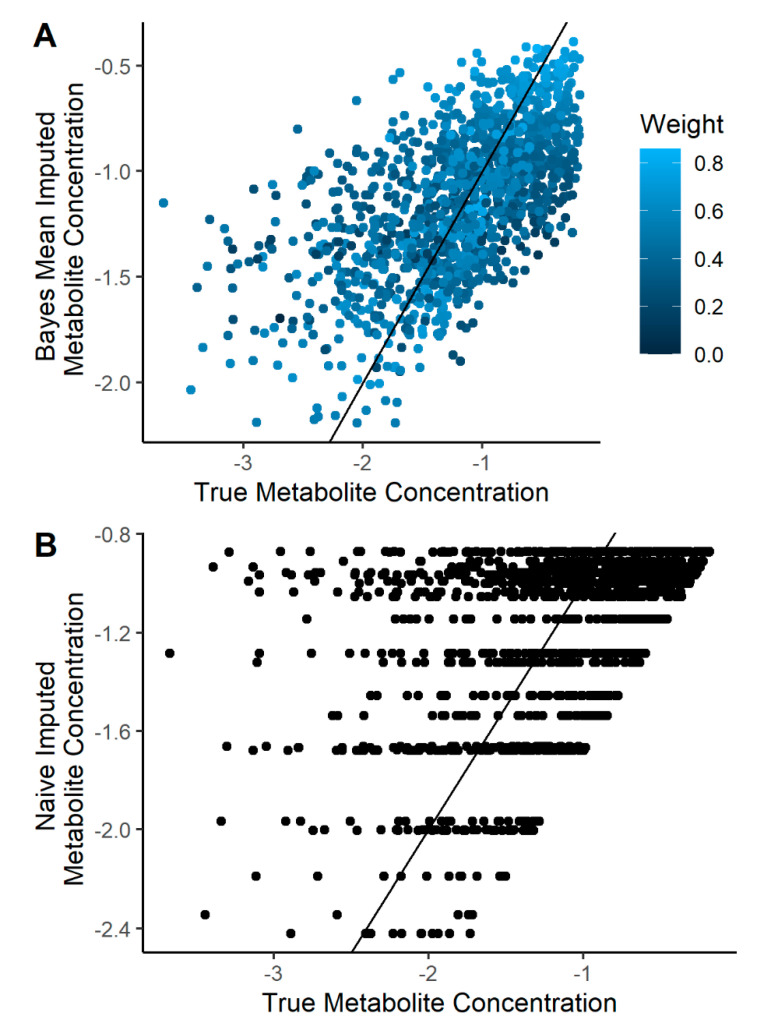
A multilevel Bayesian approach can impute the value of missing metabolite observations and capture the uncertainty of the prediction, which improves the estimation of missing data relative to a standard metabolomics approach. Missing data were introduced into the simulated metabolomics dataset at a rate of 30% and imputation was completed using a multilevel Bayesian approach (**A**) or a naïve approach (**B**). In the Bayesian approach, the mean and standard deviation per missing metabolite concentration were computed using a left-censored Bayesian regression model for each metabolite based on the top eight most correlated metabolites and a pre-defined censoring threshold. The correlation of predicted and true metabolite concentration was 0.61 but improved to 0.65 when uncertainty in the prediction was accounted for using a weight function. In the naïve approach, the missing metabolite observation was calculated as the minimum concentration for that metabolite divided by two. The correlation of predicted vs. true metabolite concentrations using the naïve approach was 0.45.

We introduced missingness into our simulated NMR dataset using a left-censor mechanism (Figure 1) with an average missing rate ranging from 0% to 30%. This parameter is the average of a scaled beta distribution and is used to control the missing value rate among each metabolite. A two-stage imputation strategy was utilized in the multilevel Bayesian model (Figure 3) and was compared to the frequentist models with a naïve imputation for each metabolite computed as the minimum concentration divided by two. The estimated metabolite concentration by Bayesian and naïve imputation was compared to the true metabolite concentration in the simulated dataset (Figure 4). The correlation between the mean Bayesian imputed and true metabolite concentrations was 0.61, compared to a correlation of 0.45 for the naïve imputation. Imputed values further from the truth tend to have a higher uncertainty. Importantly, the Bayesian regression model computes a mean (µ) and standard deviation (σ) per missing metabolite observation, where the latter is used as a proxy for the uncertainty of the models imputed prediction. Using a weighted linear regression that accounts for this uncertainty improves the correlation between Bayesian imputed and true metabolite concentrations to 0.65 (Figure 4A). Use of a multilevel Bayesian model thus allows for imputation of a missing observation based on the variance/covariance relationship of metabolites in the dataset and can model the uncertainty of the predicted value, both of which improve overall estimation of missing data.

### 2.3. Multilevel Bayesian Models Incorporating Uncertainty into Imputation Leads to Improving Effect Size Estimation in the Presence of Missing Data

**Figure 5:**
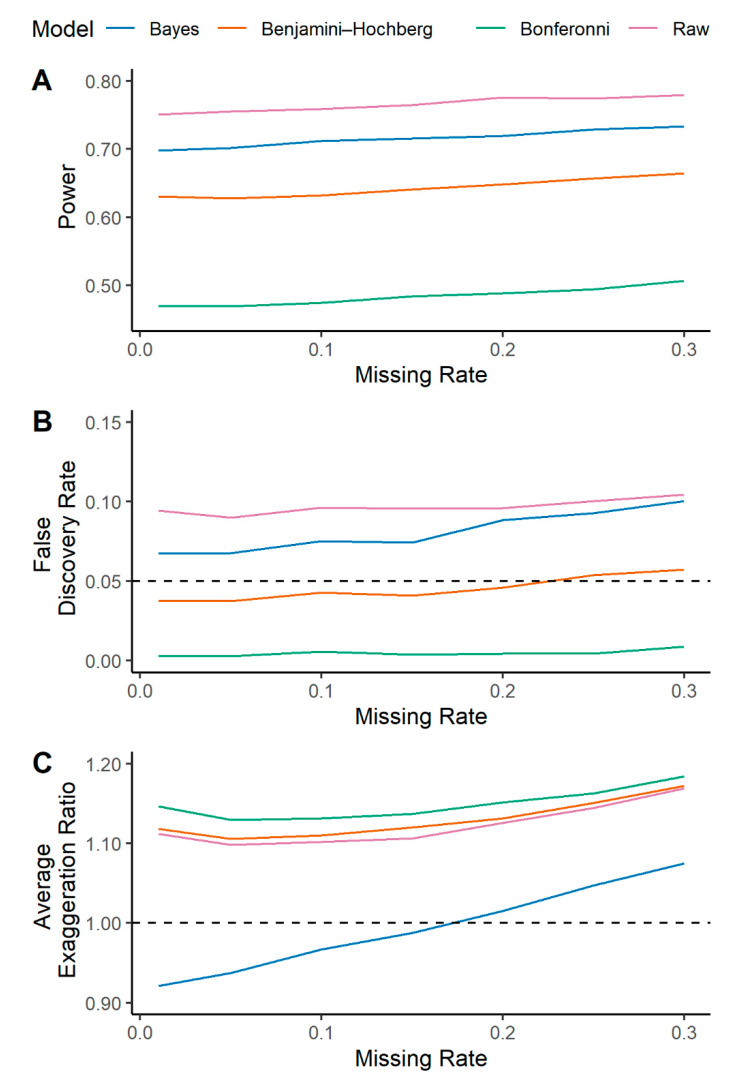
In the presence of increasing missingness in metabolomics data, a multilevel Bayesian approach offers consistent model performance while providing a more accurate estimation of metabolite effect size relative to other statistical approaches. A multilevel Bayesian model (blue lines) and a standard logistic regression (labeled raw, purple lines) were fit on a simulated metabolic dataset where 40% of metabolites were defined to be significantly different between groups (survivors vs. non-survivors). Logistic regression models were further adjusted for multiple testing according to Bonferroni (green line) and Benjamini–Hochberg (orange line). Models were fit in the presence of increasing missing data as described in the methods. Model predictions are provided as: (**A**) Power or True positive rate (TPR); (**B**) False Discovery Rate (FDR); (**C**) Average exaggeration ratio (AER) in estimated effect size. This is defined as the mean error over the set of metabolites that were significant and true (*ST*) for each model.

The power, FDR, and AER are plotted as a function of the missing rate for each model in Figure 5. As is the case for a ‘perfect’ dataset with 0% missingness, the multilevel Bayesian model has greater power relative to traditional corrections for multiple hypothesis testing (Figure 5A). The FDR is also lower in the Bayesian methodology relative to unadjusted logistic regression, and the Bonferroni correction limits FDR to the greatest degree (Figure 5B). Unsurprisingly, the AER increases as a function of increased missing rate for all models (Figure 5C). However, our Bayesian model has lower AER across all scenarios. In aggregate, the Bayesian approach limits the risk for Type M error, while maintaining a balance of power and FDR. The model performance with increasing missingness and across all simulations of sample size and fraction of significant metabolites are provided in Supplementary Table S2.

### 2.4. Application of Bayesian Models to Metabolomics Data

The multilevel Bayesian model and standard logistic regression models with or without corrections for multiple testing were fit on the original, unchanged NMR metabolomics data from patients with septic shock [19,20]. In our Bayesian model, 17/27 (63%) of the metabolites were significantly related to sepsis mortality. In the unadjusted logistic regression and logistic regression with B-H correction for FDR models, 17 (63%) were significantly related to the outcome, while only 11 (41%) were significantly related after applying a Bonferroni correction. The effect size estimates for the Bayesian approach and the standard approach (which remains constant regardless of the correction for multiple hypothesis testing) are plotted in Supplementary Figure S3. We also fit a Bayesian linear regression with *rstanarm* with a uniform prior to determine the relationship between the metabolite effect size estimated by multilevel Bayesian versus standard logistic regression. The mean of the posterior distribution for the slope estimate was 0.90, with a 95% credible interval ranging from 0.85 to 0.95. In addition, given the assumptions of the model, the probability that the slope coefficient is less than 1 is 0.9999. Since we can confidently say the slope is less than one, we can see our Bayesian model’s shrinkage of effect size estimates. All models were then applied to the original, unchanged GC-MS metabolomics dataset from exhaled breath of patients with and without acute respiratory distress syndrome (ARDS) [22]. In our multilevel Bayesian model, zero metabolites were found to be associated with a diagnosis of ARDS, while 4/42 (10%) of metabolites were associated in the unadjusted logistic regression. Upon correction for multiple testing by either Bonferroni or B-H FDR correction, a single metabolite (2%) was associated with a diagnosis of ARDS. The shrinkage of the effect size estimates in the Bayesian model relative to the frequentist models is clear with a slope estimate upon Bayesian linear regression equal to 0.34 (95% credible interval [0.32, 0.37]).

### 2.5. Prior Probability Distribution Sensitivity Analysis

Our multilevel model links logistic regression model parameters ($\beta_{xj})$ using a hierarchical prior via the parameters $\nu_{\beta x}$ (degrees freedom) and $\sigma_{\beta x}$ (scale parameter) of the t-distribution, where 0 is the mean (see methods Section 4.2). Both $\nu_{\beta x}$ and $\sigma_{\beta x}$ are learned from the data and inferred within a fully Bayesian framework. Thus, our model adapts the prior to the data and different levels of shrinkage will occur depending on the regression parameter distribution.

To determine the effect of different prior probability distributions, we compared our multilevel model with learned values for $\nu_{\beta x}$ and $\sigma_{\beta x}$ with two additional scenarios: one where $\nu_{\beta x}$ is fixed at 1 and only $\sigma_{\beta x}$ is learned, and another where $\;\nu_{\beta x}$ is fixed at 100 and $\sigma_{\beta x}$ is fixed at 5. When $\nu_{\beta x}$ is fixed at 1, then the tails of this distribution are thicker. This places greater probability around larger parameter estimates and encourages less shrinkage. When $\nu_{\beta x}$ is fixed at a large number such as 100 with a fixed $\sigma_{\beta x}$ at 5, the multilevel nature of our model is lost due to no shared parameters across regression models. The Bayesian model reduces to fitting separate regression models to each metabolite with the same, approximately normal prior. This normal prior with a large standard deviation encourages less shrinkage than the hierarchical shared prior model.

Supplementary Figure S4 shows the results of simulations under the three scenarios listed above with standard logistic regression. Relative to the fully learned multilevel model, when $\;\nu_{\beta x}$ is fixed at 1 and only $\sigma_{\beta x}$ is learned, the model has a lower FDR and a slightly improved AER. This comes at the expense of reduced power. In this situation ($\;\nu_{\beta x}$ fixed at 1), the model learned a smaller posterior value for $\sigma_{\beta x}$ with a mean (across all simulation scenarios) of 0.14 as compared to 0.29 when $\;\nu_{\beta x}$ is a free parameter. This encourages more shrinkage for smaller regression parameters and less shrinkage for large parameter values, since the tails of the t-distribution are thicker. When both $\;\nu_{\beta x}$ and $\sigma_{\beta x}$ are fixed to create a weakly informed prior (to encourage less shrinkage), the model performance is quite similar to standard frequentist logistic regression; this is consistent with the findings of others [23].

## 3. Discussion

In response to threats of scientific reproducibility and challenges to traditional statistical methodology for effect size estimation, we implemented a simulation of NMR metabolomics data (Figure 1) to evaluate the effectiveness of multilevel Bayesian models compared to standard logistic regression in a univariate variable ranking situation. We found that our Bayesian models lead to a more accurate estimation of a metabolite’s effect size and that our estimation improves upon incorporating the uncertainty of imputed missing values. These findings suggest our Bayesian methodology for metabolomics can provide more reliable results in line with the ideals of ‘New Statistics’, which supports a shift away from null hypothesis testing toward effect size estimation with uncertainty.

In the presence of complete metabolomics data (i.e., no missingness), a multilevel Bayesian model showed more accurate effect size estimation for significant metabolites (Figure 2C and Supplementary Figure S1 panel C). We then developed a two-stage imputation model (Figure 3) that appropriately models left-censored missing metabolomics data. Our imputation method provided accurate estimation of the simulated metabolite concentrations (Figure 4A) and further enhanced the accuracy of metabolite effect size estimation in regression analysis (Figure 5C). As such, our models handle missing data issues inherent to metabolomics data like other Bayesian approaches such as BayesMetab and GGSim, but further demonstrates our Bayesian models’ ability to control for Type M error as measured by the AER. This improvement is due to the multilevel model’s shrinkage and to the more accurate ‘soft’ imputation. With our 2-stage approach, researchers are not limited to our imputation method, and could use other existing methods to handle missing data [18,24,25,26,27]. However, to fully take advantage of our model, some measure of uncertainty per imputed metabolite concentration should be included.

Moreover, if a hypothesis testing framework with a null assumption of true zero effect is considered, we have shown that multilevel Bayesian modeling can control FDR and has increased power to detect true effects, regardless of the level of missing data (Figure 2A,B and Figure 5A,B). Importantly, Gelman et al. has noted that an FDR correction is most logical and useful in a scenario where there are likely to be effect sizes that are truly or nearly zero. Such an approach may be ideal in genetics and genome wide association studies, where several features are likely to have close to zero effect on a phenotype of interest. However, in targeted metabolomic investigations of highly abundant compounds, testing the null hypothesis of an effect size equal to zero may be less appropriate. Metabolites, as the end-product of biological reactions in health and disease, provide a functional, molecular phenotype of the body, which sits downstream of genetic, transcriptomic, and proteomic influence [28]. With most metabolites involved in multiple, complicated biochemical pathways, the plausibility of a truly zero effect size for a given metabolite may be quite low. Therefore, in metabolomics analysis, effect size estimation may be more useful and informative than controlling for FDR since most effect sizes may not be truly zero. Since our multilevel Bayesian model works better than standard logistic regression for controlling for Type M error, it may be more appropriate for these types of studies.

Application of our models to existing metabolomics data offers a unique opportunity to explore the benefits and limitations of the different analysis methodologies. In the NMR dataset, a multilevel Bayesian approach identified additional ‘significant’ metabolites related to the outcome of interest (sepsis mortality) compared to the conservative Bonferroni correction for multiple testing. It is therefore possible that traditional analyses of metabolomics data may be focusing on a limited pool of metabolic signatures due to the reduced power of classical methods. The relationship between multilevel Bayesian effect size estimates compared to estimates by standard logistic regression (Supplementary Figure S3) showed overall shrinkage in the Bayesian framework, as evidenced by a slope less than 1. This shrinkage may be providing more accurate estimates of significant metabolite’s true effect size based on the results of our simulations and prior research [16]. Nonetheless, there was overall agreement between effect size estimates in the two methodologies, indicating that common statistical workflows of metabolomics data analyses (e.g., removing metabolites with excessive missingness) can help guard against bias in effect size estimation.

In the GC-MS dataset, the multilevel Bayesian model did not identify any metabolites significantly related to ARDS diagnosis and produced considerable shrinkage in effect size estimation. This may represent a scenario when the fraction of significant metabolite predictors is small (e.g., less than 10%), and the multilevel Bayesian method overcorrects by estimating the σ_βx_ regularization term to be too small. Indeed, we see similar trends and overly conservative estimation in Bayesian estimated effect size in our simulations at low values for the fraction of significant metabolites (Supplementary Figure S2). However, our simulations also show that standard regression methods tend to inflate the AER, which agrees with previous reports that ‘statistically significant’ results tend to be upwardly biased in nature [3,4]. In this instance, alternative priors such as fixing $\nu_{\beta x}$ at a small value or introducing a lower bound for $\sigma_{\beta x}$ can help combat over shrinkage (Supplementary Figure S4). In the real GC-MS data, introducing a lower bound of 0.1 for $\sigma_{\beta x}$ identified a single metabolite related to ARDS, which is consistent with the Bonferroni or B-H FDR correction upon standard regression. More research will be needed to develop more effective prior distributions to prevent overcorrection and to assess model performance on untargeted metabolomic platforms.

Although our methods can be easily scaled up to include multivariate modeling, our initial focus is on the univariate metabolite ranking situation and we cannot yet speak to model performance in more complex modeling scenarios. We also did not directly compare the benefit of our approach, which incorporates the uncertainty of missing values, versus a multilevel model with strict hard imputation. Nonetheless, our ability to predict the true metabolite concentration of missing data incorporated into our simulation did improve with a weight function modeling the uncertainty in the estimate (Figure 4A). Moreover, in the simulations with increasing missing data, our models did effectively limit the increase in AER (Figure 5C); however, it did help considerably. Other important limitations include that, in situations where the researcher does not have a prior belief about the fraction of truly associated metabolites, this method may lead to overcorrection if the fraction is small (<20%). In addition, for very large datasets, with high missing rates, the Markov Chain Monte Carlo simulation approach may be time and computationally expensive.

## 4. Materials and Methods

### 4.1. Simulation Approach

To evaluate the benefit of multilevel modeling and incorporating uncertainty of imputation into downstream regression, we developed a simulation which generates data similar to real metabolomics data. We required a simulation so that we could generate a ground truth and compare how well our model works under a variety of settings. Figure 1 and Figure 3 show a schematic of our simulation approach and imputation methods, respectively. We first learned the mean and covariance parameters of a real metabolomics dataset and used this as our starting point for generating data. We then implemented a simulation that can vary the sample size per group, the fraction of significant metabolites, and the missing rate. We varied these parameters and assessed our Bayesian model and its correction mechanism with standard logistic regression using common multiple testing correction schemes. We compared the performance of these models using a variety of criteria including power, false discovery rate, and AER.

#### 4.1.1. Parameter Learning from Experimental Data

The basis for the simulation was an NMR dataset from a recent septic shock trial. In this dataset, baseline serum samples from 228 septic shock patients were quantified by ^1^H-NMR as previously described [19,20,21]. All cause 90-day mortality was 122/228 (53.5%) and 106/228 (46.5%) survived beyond 90 days. After filtering metabolites with a missing rate less than 30%, there were 27 metabolites remaining. Concentrations were log transformed and each metabolite was scaled to have a mean of zero and a standard deviation of one.

We then looked at the 90-day mark after enrollment into the trial and dichotomized patients into two groups. Those who survived and those who did not. To generate synthetic data, we modeled these data using a multivariate normal distribution where we computed a class-specific mean and covariance matrix:

(1) $$X_{i}\;\sim \;\{\begin{array}{ll} \;\mathrm{normal}(\mu_{D},\Sigma_{D}), & \mathrm{class}\left(i\right)=\mathrm{non-survivors}\\ \mathrm{normal}(\mu_{S},\Sigma_{S}),\;\; & \mathrm{class}\left(i\right)=\mathrm{survivors} \end{array}$$

Here, $\mu_{D}$ is the mean of each metabolite’s concentration and $\Sigma_{D}$ is the covariance matrix for patients who died at or before 90 days, while $\mu_{S}$ and $\Sigma_{S}$ are the mean and covariance matrix for the metabolite concentrations for patients who survived.

#### 4.1.2. Simulation Parameters

The *Number of Simulations* parameter is the number of synthetic dataset replications to generate based on the following parameters. For our analyses, we did 200 replications for each parameter combination.

The *Sample Size per Group* parameter controls the number of patients whose metabolomic profiles are simulated for survivors and non-survivors.

The *Fraction of Significant Metabolites* parameter controls the number of metabolites that are ‘truly’ different between patients who survived and those who did not. We did this by constructing a discrete probability distribution that assigns the probability that a metabolite is non-zero to be proportional to absolute difference in magnitude between survivors and non-survivors. Specifically, we assigned probabilities as follows:

(2) $$TS_{j}=\frac{\left|\;\mu_{D_{j}}-\mu_{S_{j}}\right|}{\sqrt{\frac{\sigma_{D_{j}}^{2}\;}{n_{D}}-\frac{\sigma_{S_{j}}^{2}}{n_{S}}}}$$

(3) $$p_{j}=\frac{TS_{j}}{\sum^{\;}TS_{k}}$$

The variable $TS_{j}$ is the absolute magnitude of the t-statistic between the patients who survived and those that did not for metabolite *j*, and $p_{j}$ is the probability that metabolite *j* is truly different between survivors and non-survivors. The sample sizes $n_{D}$ and $n_{S}$ are the number of samples per group for patients who are survivors and non-survivors. If *Fraction of Significant Metabolites* is equal to 0.5, we would have a sample without replacement using the $p_{j}$ probabilities until we have at least 0.5 metabolites selected. The remaining metabolites are assigned to have no true difference. We did this by creating two new mean vector variables ($\hat{\mu_{D}}$ and $\hat{\mu_{S}}$), where $\hat{\mu_{D_{j}}}=\hat{\mu_{S_{j}}}=0$ for all *j* not selected to be truly different.

The *Average Missing Rate* (*r*) parameter controls the missing rate per metabolite within the simulation. To make the distribution of missingness look similar to the real metabolomics dataset, we first fit a beta distribution to missing rate distribution of our real dataset and computed the mean $\mu_{\beta }$ and variance $\sigma_{\beta }^{2}$. Second, we scaled the mean and variance to have the target *Average Missing Rate* as follows:

(4) $$\hat{\mu_{\beta }}=r$$

(5) $$\hat{\sigma_{\beta }^{2}}=\frac{r}{\mu_{\beta }}\sigma_{\beta }^{2}$$

We then sampled a missing rate from this resulting beta distribution $m_{j}$ for metabolite *j*. This missing rate was then used to left censor the generated data. We did this by assigning all values within a quantile less than $m_{j}$ to be missing.

The final parameter was the *Maximum Missing Rate*. This parameter was set to be 0.4 in our simulation study. This parameter truncates the missing rate to be no more than the *Maximum Missing Rate*.

### 4.2. Multilevel Bayesian Logistic Regression Model

We modeled each metabolite with the outcome of interest using logistic regression with an added assumption that the regression coefficients come from a common distribution:

(6) $$\mathrm{logit}\left(p_{ij}\right)=\alpha_{j}+\beta_{xj}X_{ij}$$

The probability that patient *i* will experience the event under study for metabolite *j* is modeled by a bias parameter $\alpha_{xj}$ and the log odds ratio ($\beta_{xj}$) for metabolite *j*:

(7) $$\mathrm{logit}\left(x\right)=\frac{x}{1-x}$$

where we assume the regression coefficients for metabolites come from a common distribution centered at zero, with degrees freedom $\nu_{x}$, and a standard deviation of $\sigma_{\beta x}$:

(8) $$\beta_{xj}\sim \;\mathrm{t-distribution}\left(\nu_{\beta x},0,\sigma_{\beta x}\right).$$

The parameter $\nu_{\beta x}$ is the degrees freedom of the t-distribution and it controls the thickness of the tails. The parameter $\sigma_{\beta x}$ is the scale of the t-distribution and it controls the overall spread of the distribution of effect sizes. A smaller $\sigma_{\beta x}$ means the model will increase the shrinkage of coefficients toward zero and a smaller $\nu_{\beta x}$ will allow the potential of larger effect sizes. We assume the following distributions:

(9) $$\sigma_{\beta x}\sim {\mathrm{cauchy}(0,1)}^{+},$$

(10) $$\nu_{\beta x}\sim \;\mathrm{gamma}(2,0.1),$$

(11) $$\alpha_{xj}\sim \mathrm{normal}(0,5).$$

Here, we assume $\alpha_{xj}$ is normally distributed with a mean of zero and a standard deviation of 5. We did not perform pooling on these parameters because the primary parameters of interest are the $\beta_{xj}$ parameters. We constrained the domain of $\nu_{\beta x}$ to be at least 1.

We used Stan’s Hamiltonian Monte Carlo Markov Chain engine for parameter inference [29]. The prior parameter settings and domain for these variables follow the guidelines for weakly informed priors from the Stan user guide [30].

### 4.3. Two-Stage Imputation Model

Our two-stage imputation model that improves parameter estimation first performs a multivariate imputation within a left-censored framework to estimate the posterior distribution of missing metabolite values. This method has similarities to BayesMetab [17] and GSimp [18]; however, our purpose is to utilize the posterior of the missing values in regression analysis rather than to make a specific imputation. We then estimated the mean and the standard deviation of the missing values and fed these into a logistic regression model that operates using this uncertainty information. The result is a model that performs soft imputation and subsequently estimates parameters yielding more accurate estimates.

#### 4.3.1. Imputation Model

The input into our imputation model is a matrix of metabolite concentrations $X\in {\mathbb{R}}^{n\times m}$, an indicator matrix $Z\in {\left\{0,1\right\}}^{n\times m}$, a threshold vector $t\in R^{m}$, and a constant *c*, where *n* is the number of patients and m is the number of observations. The matrix X has a naïve imputation strategy applied to it for initiation, where we took the minimum observed value per metabolite divided by two. The entry $Z_{ij}=0$ when $X_{ij}$ is observed and $Z_{ij}=1$ when $X_{ij}$ is missing. For each metabolite, we build a regression model within a left-censored framework. The constant *c* sets the number of metabolites to use for imputing. For each metabolite, we find the *c* metabolites with the highest Pearson correlation. Specifically, we compute the likelihood $l\left(X_{ij}\right)$ as follows:

(12) $$\begin{array}{cc} l\left(X_{ij}\right) & =\{\begin{array}{cc} \mathrm{normal}\left(X_{ij},\beta_{-j}^{T}X_{i,-j}+\alpha_{j},\sigma_{j}\right), & Z_{ij}=0\\ \int_{-\infty }^{t_{j}}\mathrm{normal}{\left(a\;,\beta_{-j}^{T}X_{i,-j}+\alpha_{j},\sigma_{j}\right)}_{\;}da, & Z_{ij}=1\; \end{array} \end{array}$$

Here, the notation $\mathrm{normal}{\left(x,\mu ,\sigma \right)}_{\;}$ is the normal probability density function at point *x*, with mean μ, and standard deviation σ. The vector $\beta_{-j}^{T}\in {\mathbb{R}}^{c\times 1}$ is a vector of regression coefficients for metabolite j. The −j notation indicates that the metabolite j is not used in for imputing itself. The vector $X_{i,-j}\in {\mathbb{R}}^{c\times 1}$ is the vector of metabolite concentrations for the *c* metabolites most correlated with metabolite j. The parameter $\alpha_{j}$ is the intercept parameter for the imputation equation for metabolite j. In words, the likelihood of $X_{ij}$ is computed using a normal probability density function when $X_{ij}$ is observed. However, when $X_{ij}$ is missing, we compute the normal cumulative distribution function which averages the normal probability density function from −∞ to left-censoring threshold $t_{j}$ for metabolite j. By using the normal cumulative distribution function, we are not biasing the regression coefficient estimates as we would by making a hard imputation using a less informed method.

The priors for the parameters of this model are as follows:

(13) $$\beta_{-j}^{T}\;\sim \;\mathrm{normal}\left(0,\sigma_{\beta_{-j}}\;\right)$$

(14) $$\sigma_{\beta_{-j}}\;\sim \;\mathrm{normal}\left(0,1\right)$$

(15) $$\alpha_{j}\;\sim \;\mathrm{uniform}\left(-\infty ,\infty \right)$$

(16) $$\sigma_{j}\;\sim \;\mathrm{uniform}\left(0,\infty \right)$$

The parameters $\beta_{-j}^{T}$ come from a shared prior distribution with standard deviation $\sigma_{\beta_{-j}}$. This provides a pooled shrinkage across all metabolites and will have a similar effect as ridge regression.

After fitting this model, we computed the uncensored mean $E_{ij}$ and standard deviation $S_{ij}$ using the posterior of each missing metabolite value.

#### 4.3.2. Logistic Regression with Uncertainty

This model is very similar to the model in Section 4.3 with the added ability to sample from right truncated normal distributions with mean $E_{ij}$ and standard deviation $S_{ij}$, when $Z_{ij}=1$:

(17) $${\hat{X}}_{ij}=\{\begin{array}{cc} X_{ij}, & Z_{ij}=0\\ X_{ij}\;{\sim \;\mathrm{normal}\left(E_{ij},\;S_{ij}\right)|}_{-\infty }^{t_{j}}, & Z_{ij}=1 \end{array}$$

In other words, we defined a variable ${\hat{X}}_{ij}$ which is equal to $X_{ij}$ when $X_{ij}$ is observed and ${\hat{X}}_{ij}$ is equal to a sample from the right truncated distribution centered at $E_{ij}$ with standard deviation $S_{ij}$. These parameters were computed using the model presented in 4.3.1 and represent the mean and standard deviation of the posterior distribution for the missing metabolite value in metabolite j for sample i. The notation ${X_{ij}\sim \;\mathrm{normal}\left(E_{ij},\;S_{ij}\right)|}_{-\infty }^{t_{j}}$ states that $X_{ij}$ is sampled from the right truncated normal distribution whose domain is $\left(-\infty ,\;\;t_{j}\right]$.

We then modify the regression equation as follows:

(18) $$\mathrm{logit}\left(p_{ij}\right)=\alpha_{j}+\beta_{xj}{\hat{X}}_{ij}.$$

The result is a logistic regression model that operates on samples from the missing values posterior distribution. This adds uncertainty to the model and leads to more accurate parameter estimates provided the imputed posterior distribution is closer to the truth unobserved value than a naïve imputation.

#### 4.3.3. Method Comparison

We compared our Bayesian hierarchical model to standard logistic regression in the following ways. First, we applied three forms of correction to standard logistic regression: No corrections, Bonferroni correction, and B-H FDR correction. We applied these *p*-value correction approaches and required the adjusted *p*-value to be less than 0.05 to be declared significant. For our Bayesian model, we declared a metabolite *j* to be significant if the probability that $\beta_{xj}<0$ is greater than 0.975 or the probability that $\beta_{xj}>0$ is greater than 0.975. In other words, the tail probability of either tail of the posterior of $\beta_{xj}$ must be at least 0.975.

We then used the following metrics to compare these multiple testing correction strategies: false discovery rate $\left(\frac{FP}{TP+FP}\right)$, power $\left(\frac{TP}{TP+FN}\right)$, and the AER in estimated effect size. Here, TP is the number of true positives (those declared to be significant and were significant), FP is the number of false positives (those declared to be positive but were negative), and FN is the number of false negatives (those declared to be negative but were actually positive). The AER in estimated effect size was computed as follows over the set of metabolites that were significant and true (*ST*) for each model, where |ST| is the cardinality of (or the number of elements in) ST) [5]:

(19) $$AER=\frac{1}{\left|ST\right|}\underset{j\in ST}{\sum }\left|\frac{\beta_{xj}^{*}}{\beta_{xj}}\right|$$

The parameter $\beta_{xj}^{*}\;$ corresponds to estimated effect size for metabolite *j* and $\beta_{xj}$ was the true effect size. For our Bayesian Models, we took $\beta_{xj}^{*}\;$ as the mean of the posterior distribution. All of these criteria were averaged over the number of *Number of Simulations*.

### 4.4. Imputation Quality Evaluation

To evaluate the quality of our imputation model worked and the benefit of including uncertainty into the logistic regression, we did the following: first, we plotted the uncensored values using the simulated data against the imputed dataset when the *Average Missing Rate* was 0.3. We colored the points using a weight ($w_{ij}$) for patient *i* for metabolite *j* using the posterior standard deviation of the missing metabolite concentrations ($\sigma_{ij}$):

(20) $$w_{ij}=1-\frac{\sigma_{ij}}{\max\left(\sigma_{ij}\right)}.$$

This function gives higher weight to metabolites with less uncertainty and less weight to samples with higher uncertainty. We also applied this weight function to a simple linear regression function and computed the correlation between the true missing concentrations and the imputed missing concentrations with and without the weight function.

### 4.5. Real Data Comparison

We then applied the standard regression approach and our Bayesian model to the real NMR dataset of septic shock with 228 samples and 27 metabolites after filtering [19,20] a GC-MS dataset from patients with and without ARDS with 85 samples and 79 metabolites [22]. For these datasets, we compared the number of significant metabolites predicted by each model and we compared the estimated effect size of metabolites. We did this comparison by correlating the effect sizes estimated from our Bayesian model with the effect sizes using standard logistic regression. Using *rstanarm* version 2.19.3 [31], we constructed a linear model with the Bayesian model’s effect size as the dependent variable and the standard logistic regression as the independent variable. If there were no difference in effect sizes, then the slope of this model would be one. We computed the 95% credible interval from the posterior from this linear model and compared this to the expected value under the null.

## 5. Conclusions

In conclusion, we assessed if a multilevel model could simultaneously adjust for multiple testing and offer improved effect size estimation. Our simulations of NMR metabolomics data showed that a multilevel Bayesian model offers greater power and mitigates the risk of false discovery compared to standard regression methods. In addition, our Bayesian method more accurately estimated the effect size of significant metabolites, limiting Type M error. We then asked the novel question as to whether incorporating imputation uncertainty would further improve effect size estimation. Our multilevel Bayesian model easily allows us to not only estimate the concentration of missing values, but also incorporate the uncertainty of this estimate, which further improved effect size estimation. Therefore, analysts should consider multilevel Bayesian models for more accurate effect size estimation for metabolomics data.

## Data Availability

The NMR metabolomics data used in this work is available at the NIH Metabolomics Workbench: https://www.metabolomicsworkbench.org/doi:10.21228/M8VX0Z. The computer code used to generate the simulations, statistical models, and data visualization is available on Github: https://github.com/MCIRCC/multilevel-bayesian-metabolomics.

## Supplementary Materials

The following are available online at https://www.mdpi.com/2218-1989/10/8/319/s1, Figure S1: A multilevel Bayesian approach offers increased power and controls for false discovery while providing a more accurate estimation of metabolite effect size relative to other statistical correction approaches, Figure S2: Comparison of model prediction performance as a function of the fraction of significant metabolite predictors, Figure S3: Effect size estimates in real metabolomics data by a multilevel Bayesian model compared to a standard or frequentist logistic regression, Figure S4: Altering the prior probability distribution can affect model performance, Table S1: Model performance across varying simulation parameters, Table S2: Model performance across varying simulation parameters in the presence of missing data.
