# Supplementary material for: A Multilevel Bayesian Approach to Improve Effect Size Estimation in Regression Modeling of Metabolomics Data Utilizing Imputation with Uncertainty
Source: Metabolites. 2020 Aug 6;10(8):319. doi: 10.3390/metabo10080319 (PMC7465156; doi:10.3390/metabo10080319)

Figure S1: **A multilevel Bayesian approach offers increased power and controls for false discovery while providing a more accurate estimation of metabolite effect size relative to other statistical correction approaches.** A multilevel Bayesian model (blue lines) and a standard logistic regression (labeled raw, purple lines) were fit on a simulated metabolic dataset where 70% of metabolites were defined to be significantly different between groups (survivors vs. non-survivors). Logistic regression models were further adjusted for multiple testing according to Bonferroni (green line) and Benjamini–Hochberg (orange line). Models were fit at different sample sizes per group without the presence of missing data as described in the methods. Model predictions are provided as: (A) Power or True positive rate (TPR); (B) False Discovery Rate (FDR); (C) Average exaggeration ratio (AER) in estimated effect size. This is defined as the mean error over the set of metabolites that were significant and true (*ST*) for each model.


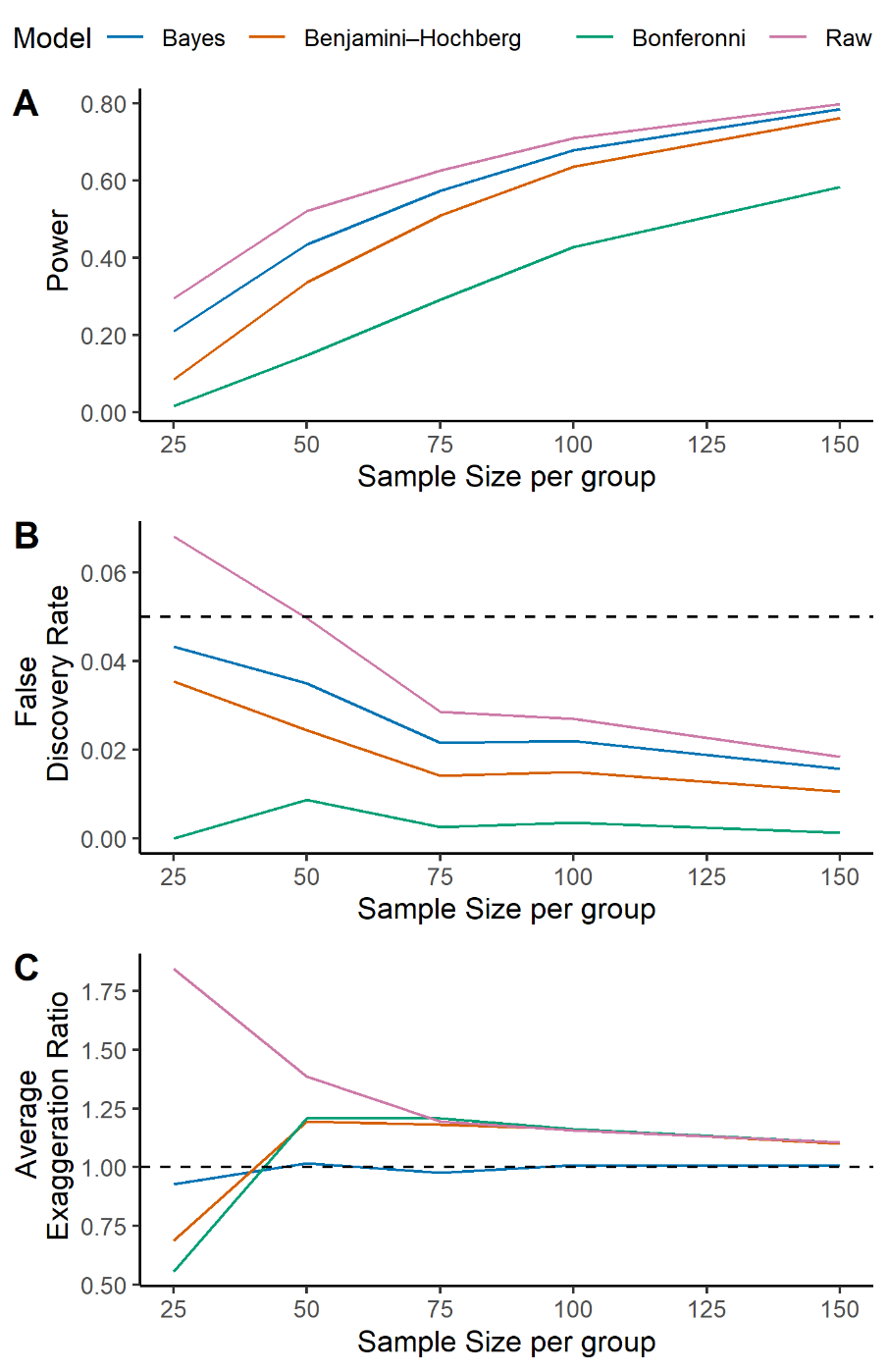


Figure S2: **Comparison of model prediction performance as a function of the fraction of significant metabolite predictors.** A multilevel Bayesian model (blue lines) and a standard logistic regression (labeled raw, purple lines) were fit on a simulated metabolic dataset where the percent of metabolites that were significantly different between groups (survivors vs. non-survivors) varied between 10% and 80%. Logistic regression models were further adjusted for multiple testing according to Bonferroni (green line) and Benjamini–Hochberg (orange line). Models were fit at a set sample size of 150 patients per group in the presence of no missing data. Model predictions are provided as: (A) Power or True positive rate (TPR); (B) False Discovery Rate (FDR); (C) Average exaggeration ratio (AER) in estimated effect size. This is defined as the mean error over the set of metabolites that were significant and true (*ST*) for each model.


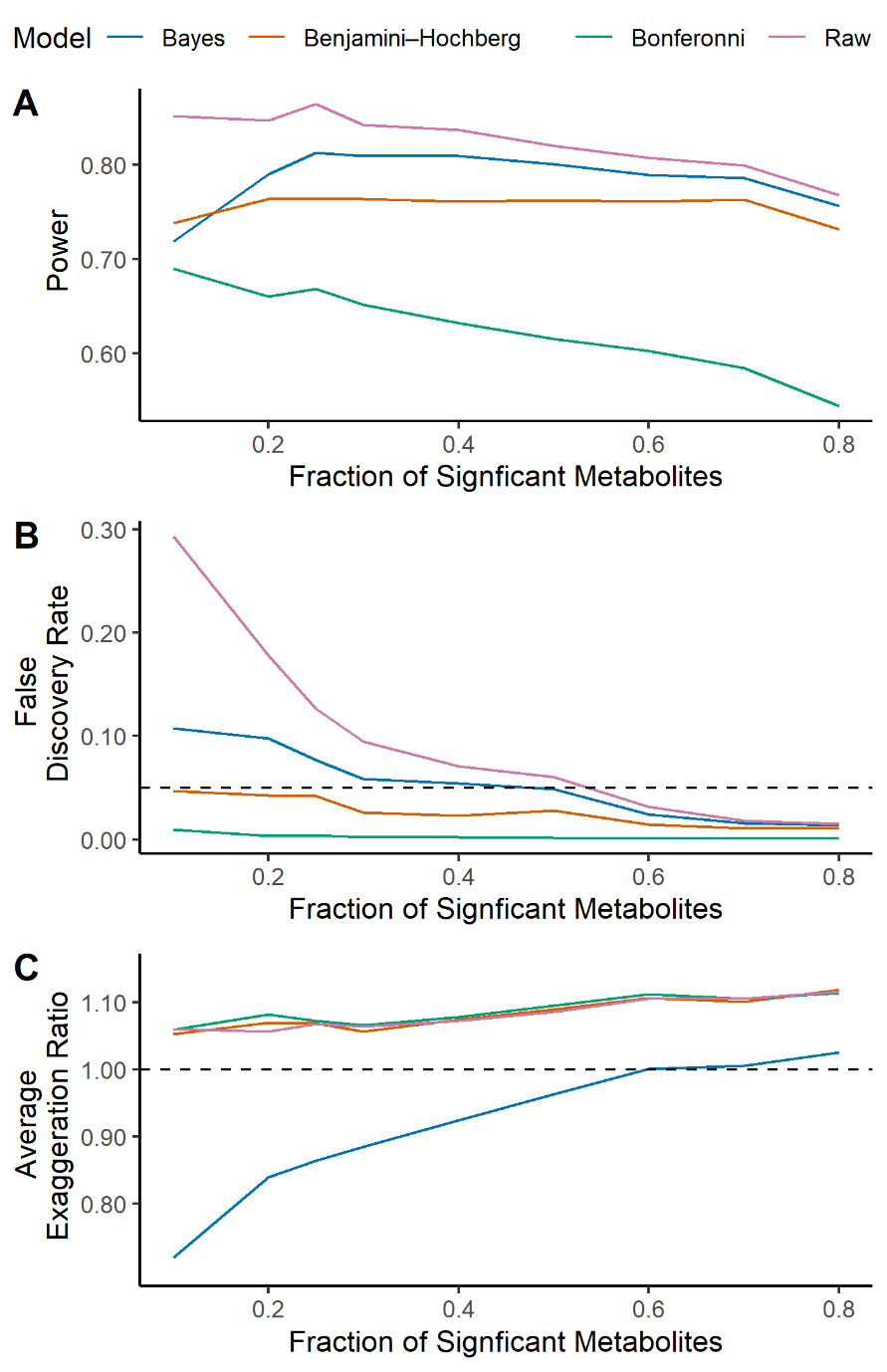


Figure S3: **Effect size estimates in real metabolomics data by a multilevel Bayesian model compared to a standard or frequentist logistic regression**. Effect size estimates for 27 metabolites measured by ^1^H-NMR in patients with septic shock. Metabolites were tested in a univariate fashion for an association with 90-day mortality. Metabolites with a significant association by Bayesian analysis were considered. Note, zero metabolites measured by GC-MS in patients with and without acute respiratory distress syndrome were predicted to be significant by Bayesian analysis and are thus not included in this model.


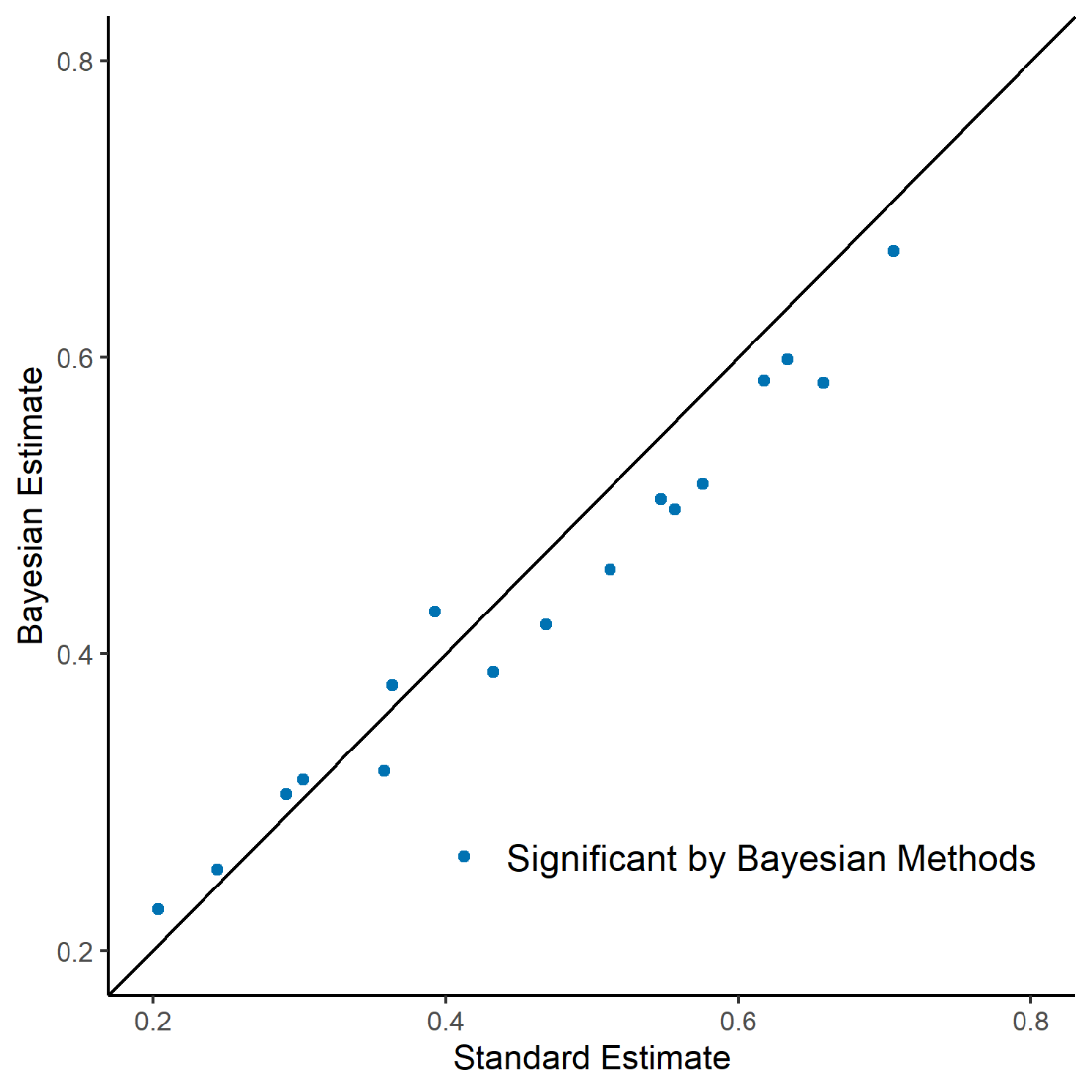


Figure S4: **Altering the prior probability distribution can affect model performance.**  Bayesian models with different prior probability distributions and a standard logistic were fit on a simulated metabolic dataset where 40% of metabolites were defined to be significantly different between groups (survivors vs. non-survivors). The blue lines represent a multilevel model where both $\nu_{\beta x}$ and $\sigma_{\beta x}$ are learned from the data and inferred within a fully Bayesian framework. Please note this is consistent with the modeling framework labeled as ‘Bayes’ in other figures. The orange line represents a scenario where $\nu_{\beta x}$ is set to be 1 and only $\sigma_{\beta x}$ is learned. The green line represents a very weakly informed Bayesian approach where $\nu_{\beta x}$ is set to 100 and $\sigma_{\beta x}$ to 5. This approximates a wide normal distribution and allows for a wide range of parameter values. The purple line represents a standard logistic regression and is consistent with the modeling framework labeled as ‘Raw’ in other figures. Models were fit at different sample sizes per group without the presence of missing data as described in the methods. Model predictions are provided as: (A) Power or True positive rate (TPR); (B) False Discovery Rate (FDR); (C) Average exaggeration ratio (AER) in estimated effect size. This is defined as the mean error over the set of metabolites that were significant and true (*ST*) for each model.


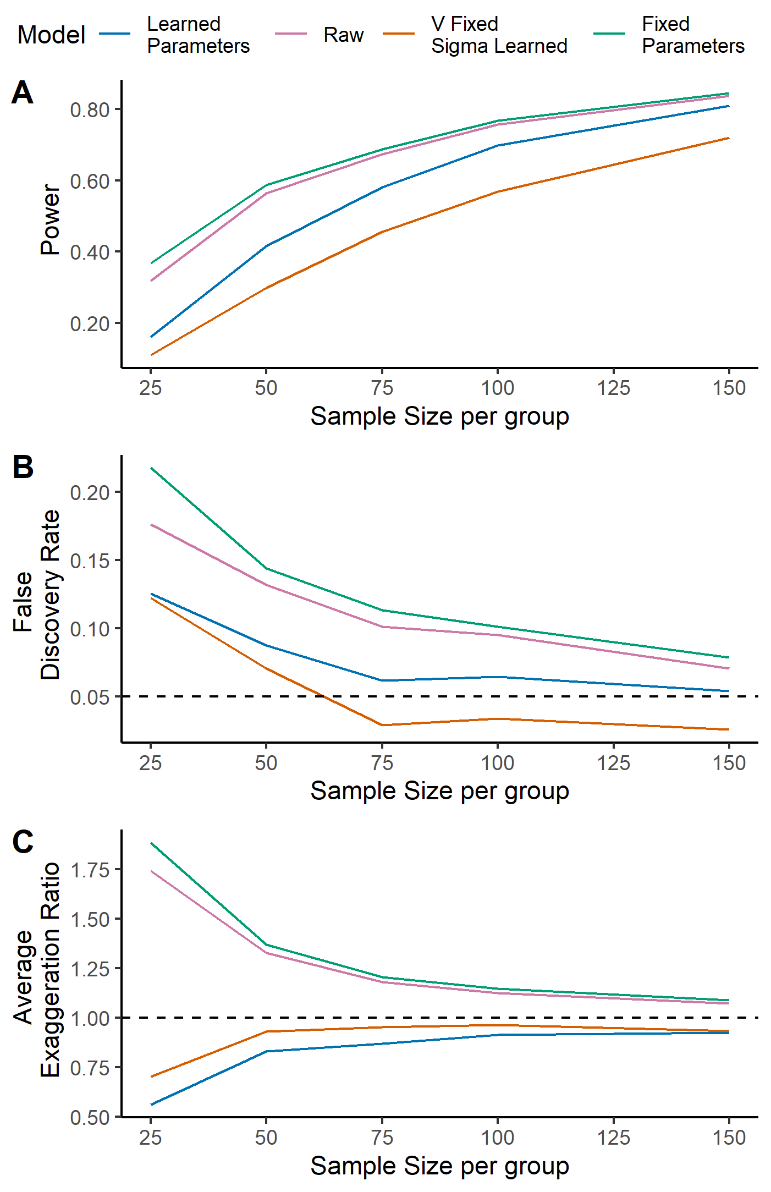

Supplement: Supplementary file 1 [file metabolites-10-00319-s001.zip › Gillies_metabolites-852665_SupplementaryFigures_CleanCopy.docx]
