# Supplementary material for: A Multilevel Bayesian Approach to Improve Effect Size Estimation in Regression Modeling of Metabolomics Data Utilizing Imputation with Uncertainty
Source: Metabolites. 2020 Aug 6;10(8):319. doi: 10.3390/metabo10080319 (PMC7465156; doi:10.3390/metabo10080319)
Supplement: Supplementary file 1 [file metabolites-10-00319-s001.zip › Table S1.docx]

**Table S1:** Model performance across varying simulation parameters.

| ***Model***  ***Method***^ | ***Fraction of Significant Metabolites*** | ***Sample Size***  ***per Group*** | ***True Positives*** | ***False Negatives*** | ***False***  ***Positives*** | ***True***  ***Negatives*** | ***Average Exaggeration Ratio*** | ***FDR^*^*** | ***Power***^†^ |
| --- | --- | --- | --- | --- | --- | --- | --- | --- | --- |
| Bayesian | 0.1 | 25 | 50 | 550 | 30 | 4770 | 0.189 | 0.375 | 0.083 |
| B-H | 0.1 | 25 | 17 | 583 | 1 | 4799 | 0.207 | 0.056 | 0.028 |
| Bonferroni | 0.1 | 25 | 15 | 585 | 1 | 4799 | 0.197 | 0.063 | 0.025 |
| Raw | 0.1 | 25 | 214 | 386 | 211 | 4589 | 1.305 | 0.496 | 0.357 |
| Bayesian | 0.1 | 50 | 132 | 468 | 71 | 4729 | 0.320 | 0.350 | 0.220 |
| B-H | 0.1 | 50 | 144 | 456 | 22 | 4778 | 0.727 | 0.133 | 0.240 |
| Bonferroni | 0.1 | 50 | 118 | 482 | 5 | 4795 | 0.708 | 0.041 | 0.197 |
| Raw | 0.1 | 50 | 367 | 233 | 253 | 4547 | 1.266 | 0.408 | 0.612 |
| Bayesian | 0.1 | 75 | 199 | 401 | 79 | 4721 | 0.412 | 0.284 | 0.332 |
| B-H | 0.1 | 75 | 264 | 336 | 26 | 4774 | 0.925 | 0.090 | 0.440 |
| Bonferroni | 0.1 | 75 | 219 | 381 | 6 | 4794 | 0.895 | 0.027 | 0.365 |
| Raw | 0.1 | 75 | 436 | 164 | 227 | 4573 | 1.145 | 0.342 | 0.727 |
| Bayesian | 0.1 | 100 | 316 | 284 | 70 | 4730 | 0.590 | 0.181 | 0.527 |
| B-H | 0.1 | 100 | 348 | 252 | 34 | 4766 | 1.026 | 0.089 | 0.580 |
| Bonferroni | 0.1 | 100 | 312 | 288 | 5 | 4795 | 1.020 | 0.016 | 0.520 |
| Raw | 0.1 | 100 | 482 | 118 | 253 | 4547 | 1.117 | 0.344 | 0.803 |
| Bayesian | 0.1 | 150 | 431 | 169 | 52 | 4748 | 0.720 | 0.108 | 0.718 |
| B-H | 0.1 | 150 | 443 | 157 | 22 | 4778 | 1.053 | 0.047 | 0.738 |
| Bonferroni | 0.1 | 150 | 414 | 186 | 4 | 4796 | 1.060 | 0.010 | 0.690 |
| Raw | 0.1 | 150 | 511 | 89 | 212 | 4588 | 1.061 | 0.293 | 0.852 |
| Bayesian | 0.2 | 25 | 132 | 1068 | 29 | 4171 | 0.338 | 0.180 | 0.110 |
| B-H | 0.2 | 25 | 50 | 1150 | 2 | 4198 | 0.339 | 0.038 | 0.042 |
| Bonferroni | 0.2 | 25 | 24 | 1176 | 1 | 4199 | 0.295 | 0.040 | 0.020 |
| Raw | 0.2 | 25 | 419 | 781 | 154 | 4046 | 1.569 | 0.269 | 0.349 |
| Bayesian | 0.2 | 50 | 432 | 768 | 58 | 4142 | 0.665 | 0.118 | 0.360 |
| B-H | 0.2 | 50 | 392 | 808 | 19 | 4181 | 1.077 | 0.046 | 0.327 |
| Bonferroni | 0.2 | 50 | 268 | 932 | 4 | 4196 | 1.057 | 0.015 | 0.223 |
| Raw | 0.2 | 50 | 741 | 459 | 185 | 4015 | 1.318 | 0.200 | 0.618 |
| Bayesian | 0.2 | 75 | 691 | 509 | 103 | 4097 | 0.775 | 0.130 | 0.576 |
| B-H | 0.2 | 75 | 644 | 556 | 45 | 4155 | 1.139 | 0.065 | 0.537 |
| Bonferroni | 0.2 | 75 | 497 | 703 | 9 | 4191 | 1.142 | 0.018 | 0.414 |
| Raw | 0.2 | 75 | 896 | 304 | 233 | 3967 | 1.201 | 0.206 | 0.747 |
| Bayesian | 0.2 | 100 | 809 | 391 | 94 | 4106 | 0.772 | 0.104 | 0.674 |
| B-H | 0.2 | 100 | 742 | 458 | 49 | 4151 | 1.093 | 0.062 | 0.618 |
| Bonferroni | 0.2 | 100 | 592 | 608 | 10 | 4190 | 1.111 | 0.017 | 0.493 |
| Raw | 0.2 | 100 | 939 | 261 | 222 | 3978 | 1.111 | 0.191 | 0.783 |
| Bayesian | 0.2 | 150 | 948 | 252 | 103 | 4097 | 0.840 | 0.098 | 0.790 |
| B-H | 0.2 | 150 | 916 | 284 | 41 | 4159 | 1.070 | 0.043 | 0.763 |
| Bonferroni | 0.2 | 150 | 792 | 408 | 3 | 4197 | 1.083 | 0.004 | 0.660 |
| Raw | 0.2 | 150 | 1016 | 184 | 220 | 3980 | 1.057 | 0.178 | 0.847 |
| Bayesian | 0.25 | 25 | 191 | 1209 | 59 | 3941 | 0.387 | 0.236 | 0.136 |
| B-H | 0.25 | 25 | 71 | 1329 | 3 | 3997 | 0.413 | 0.041 | 0.051 |
| Bonferroni | 0.25 | 25 | 36 | 1364 | 0 | 4000 | 0.358 | 0.000 | 0.026 |
| Raw | 0.25 | 25 | 477 | 923 | 188 | 3812 | 1.537 | 0.283 | 0.341 |
| Bayesian | 0.25 | 50 | 525 | 875 | 98 | 3902 | 0.705 | 0.157 | 0.375 |
| B-H | 0.25 | 50 | 431 | 969 | 37 | 3963 | 1.105 | 0.079 | 0.308 |
| Bonferroni | 0.25 | 50 | 273 | 1127 | 6 | 3994 | 1.027 | 0.022 | 0.195 |
| Raw | 0.25 | 50 | 799 | 601 | 266 | 3734 | 1.397 | 0.250 | 0.571 |
| Bayesian | 0.25 | 75 | 741 | 659 | 87 | 3913 | 0.756 | 0.105 | 0.529 |
| B-H | 0.25 | 75 | 682 | 718 | 29 | 3971 | 1.091 | 0.041 | 0.487 |
| Bonferroni | 0.25 | 75 | 499 | 901 | 6 | 3994 | 1.099 | 0.012 | 0.356 |
| Raw | 0.25 | 75 | 962 | 438 | 220 | 3780 | 1.130 | 0.186 | 0.687 |
| Bayesian | 0.25 | 100 | 954 | 446 | 107 | 3893 | 0.830 | 0.101 | 0.681 |
| B-H | 0.25 | 100 | 891 | 509 | 50 | 3950 | 1.123 | 0.053 | 0.636 |
| Bonferroni | 0.25 | 100 | 706 | 694 | 3 | 3997 | 1.129 | 0.004 | 0.504 |
| Raw | 0.25 | 100 | 1085 | 315 | 202 | 3798 | 1.114 | 0.157 | 0.775 |
| Bayesian | 0.25 | 150 | 1138 | 262 | 95 | 3905 | 0.864 | 0.077 | 0.813 |
| B-H | 0.25 | 150 | 1070 | 330 | 47 | 3953 | 1.069 | 0.042 | 0.764 |
| Bonferroni | 0.25 | 150 | 936 | 464 | 4 | 3996 | 1.072 | 0.004 | 0.669 |
| Raw | 0.25 | 150 | 1210 | 190 | 175 | 3825 | 1.068 | 0.126 | 0.864 |
| Bayesian | 0.3 | 25 | 270 | 1530 | 44 | 3556 | 0.495 | 0.140 | 0.150 |
| B-H | 0.3 | 25 | 84 | 1716 | 9 | 3591 | 0.365 | 0.097 | 0.047 |
| Bonferroni | 0.3 | 25 | 32 | 1768 | 1 | 3599 | 0.339 | 0.030 | 0.018 |
| Raw | 0.3 | 25 | 584 | 1216 | 164 | 3436 | 1.654 | 0.219 | 0.324 |
| Bayesian | 0.3 | 50 | 670 | 1130 | 80 | 3520 | 0.728 | 0.107 | 0.372 |
| B-H | 0.3 | 50 | 536 | 1264 | 38 | 3562 | 1.006 | 0.066 | 0.298 |
| Bonferroni | 0.3 | 50 | 311 | 1489 | 4 | 3596 | 0.990 | 0.013 | 0.173 |
| Raw | 0.3 | 50 | 1012 | 788 | 195 | 3405 | 1.292 | 0.162 | 0.562 |
| Bayesian | 0.3 | 75 | 1016 | 784 | 85 | 3515 | 0.827 | 0.077 | 0.564 |
| B-H | 0.3 | 75 | 883 | 917 | 33 | 3567 | 1.133 | 0.036 | 0.491 |
| Bonferroni | 0.3 | 75 | 641 | 1159 | 4 | 3596 | 1.143 | 0.006 | 0.356 |
| Raw | 0.3 | 75 | 1223 | 577 | 167 | 3433 | 1.153 | 0.120 | 0.679 |
| Bayesian | 0.3 | 100 | 1212 | 588 | 110 | 3490 | 0.868 | 0.083 | 0.673 |
| B-H | 0.3 | 100 | 1130 | 670 | 56 | 3544 | 1.126 | 0.047 | 0.628 |
| Bonferroni | 0.3 | 100 | 870 | 930 | 7 | 3593 | 1.133 | 0.008 | 0.483 |
| Raw | 0.3 | 100 | 1377 | 423 | 184 | 3416 | 1.109 | 0.118 | 0.765 |
| Bayesian | 0.3 | 150 | 1457 | 343 | 91 | 3509 | 0.885 | 0.059 | 0.809 |
| B-H | 0.3 | 150 | 1374 | 426 | 37 | 3563 | 1.056 | 0.026 | 0.763 |
| Bonferroni | 0.3 | 150 | 1173 | 627 | 3 | 3597 | 1.066 | 0.003 | 0.652 |
| Raw | 0.3 | 150 | 1516 | 284 | 159 | 3441 | 1.064 | 0.095 | 0.842 |
| Bayesian | 0.4 | 25 | 355 | 1845 | 51 | 3149 | 0.562 | 0.126 | 0.161 |
| B-H | 0.4 | 25 | 146 | 2054 | 9 | 3191 | 0.506 | 0.058 | 0.066 |
| Bonferroni | 0.4 | 25 | 49 | 2151 | 1 | 3199 | 0.466 | 0.020 | 0.022 |
| Raw | 0.4 | 25 | 702 | 1498 | 150 | 3050 | 1.743 | 0.176 | 0.319 |
| Bayesian | 0.4 | 50 | 916 | 1284 | 88 | 3112 | 0.831 | 0.088 | 0.416 |
| B-H | 0.4 | 50 | 719 | 1481 | 49 | 3151 | 1.149 | 0.064 | 0.327 |
| Bonferroni | 0.4 | 50 | 386 | 1814 | 2 | 3198 | 1.097 | 0.005 | 0.175 |
| Raw | 0.4 | 50 | 1239 | 961 | 188 | 3012 | 1.327 | 0.132 | 0.563 |
| Bayesian | 0.4 | 75 | 1277 | 923 | 84 | 3116 | 0.869 | 0.062 | 0.580 |
| B-H | 0.4 | 75 | 1125 | 1075 | 41 | 3159 | 1.142 | 0.035 | 0.511 |
| Bonferroni | 0.4 | 75 | 733 | 1467 | 3 | 3197 | 1.137 | 0.004 | 0.333 |
| Raw | 0.4 | 75 | 1480 | 720 | 167 | 3033 | 1.180 | 0.101 | 0.673 |
| Bayesian | 0.4 | 100 | 1534 | 666 | 106 | 3094 | 0.912 | 0.065 | 0.697 |
| B-H | 0.4 | 100 | 1398 | 802 | 58 | 3142 | 1.132 | 0.040 | 0.635 |
| Bonferroni | 0.4 | 100 | 1042 | 1158 | 3 | 3197 | 1.154 | 0.003 | 0.474 |
| Raw | 0.4 | 100 | 1664 | 536 | 175 | 3025 | 1.125 | 0.095 | 0.756 |
| Bayesian | 0.4 | 150 | 1780 | 420 | 102 | 3098 | 0.925 | 0.054 | 0.809 |
| B-H | 0.4 | 150 | 1674 | 526 | 40 | 3160 | 1.074 | 0.023 | 0.761 |
| Bonferroni | 0.4 | 150 | 1391 | 809 | 3 | 3197 | 1.078 | 0.002 | 0.632 |
| Raw | 0.4 | 150 | 1841 | 359 | 140 | 3060 | 1.072 | 0.071 | 0.837 |
| Bayesian | 0.5 | 25 | 481 | 2319 | 31 | 2569 | 0.668 | 0.061 | 0.172 |
| B-H | 0.5 | 25 | 144 | 2656 | 7 | 2593 | 0.479 | 0.046 | 0.051 |
| Bonferroni | 0.5 | 25 | 47 | 2753 | 1 | 2599 | 0.414 | 0.021 | 0.017 |
| Raw | 0.5 | 25 | 857 | 1943 | 117 | 2483 | 1.768 | 0.120 | 0.306 |
| Bayesian | 0.5 | 50 | 1246 | 1554 | 65 | 2535 | 0.946 | 0.050 | 0.445 |
| B-H | 0.5 | 50 | 1000 | 1800 | 26 | 2574 | 1.219 | 0.025 | 0.357 |
| Bonferroni | 0.5 | 50 | 496 | 2304 | 2 | 2598 | 1.205 | 0.004 | 0.177 |
| Raw | 0.5 | 50 | 1546 | 1254 | 141 | 2459 | 1.323 | 0.084 | 0.552 |
| Bayesian | 0.5 | 75 | 1705 | 1095 | 107 | 2493 | 0.991 | 0.059 | 0.609 |
| B-H | 0.5 | 75 | 1503 | 1297 | 60 | 2540 | 1.217 | 0.038 | 0.537 |
| Bonferroni | 0.5 | 75 | 962 | 1838 | 2 | 2598 | 1.262 | 0.002 | 0.344 |
| Raw | 0.5 | 75 | 1872 | 928 | 169 | 2431 | 1.225 | 0.083 | 0.669 |
| Bayesian | 0.5 | 100 | 1935 | 865 | 77 | 2523 | 0.940 | 0.038 | 0.691 |
| B-H | 0.5 | 100 | 1778 | 1022 | 52 | 2548 | 1.129 | 0.028 | 0.635 |
| Bonferroni | 0.5 | 100 | 1248 | 1552 | 7 | 2593 | 1.154 | 0.006 | 0.446 |
| Raw | 0.5 | 100 | 2081 | 719 | 126 | 2474 | 1.118 | 0.057 | 0.743 |
| Bayesian | 0.5 | 150 | 2241 | 559 | 115 | 2485 | 0.964 | 0.049 | 0.800 |
| B-H | 0.5 | 150 | 2135 | 665 | 62 | 2538 | 1.090 | 0.028 | 0.763 |
| Bonferroni | 0.5 | 150 | 1723 | 1077 | 3 | 2597 | 1.095 | 0.002 | 0.615 |
| Raw | 0.5 | 150 | 2295 | 505 | 148 | 2452 | 1.086 | 0.061 | 0.820 |
| Bayesian | 0.6 | 25 | 641 | 2759 | 34 | 1966 | 0.752 | 0.050 | 0.189 |
| B-H | 0.6 | 25 | 232 | 3168 | 7 | 1993 | 0.548 | 0.029 | 0.068 |
| Bonferroni | 0.6 | 25 | 61 | 3339 | 1 | 1999 | 0.521 | 0.016 | 0.018 |
| Raw | 0.6 | 25 | 995 | 2405 | 99 | 1901 | 1.816 | 0.090 | 0.293 |
| Bayesian | 0.6 | 50 | 1442 | 1958 | 56 | 1944 | 0.953 | 0.037 | 0.424 |
| B-H | 0.6 | 50 | 1161 | 2239 | 26 | 1974 | 1.226 | 0.022 | 0.341 |
| Bonferroni | 0.6 | 50 | 529 | 2871 | 1 | 1999 | 1.220 | 0.002 | 0.156 |
| Raw | 0.6 | 50 | 1754 | 1646 | 112 | 1888 | 1.320 | 0.060 | 0.516 |
| Bayesian | 0.6 | 75 | 1962 | 1438 | 52 | 1948 | 0.934 | 0.026 | 0.577 |
| B-H | 0.6 | 75 | 1706 | 1694 | 28 | 1972 | 1.148 | 0.016 | 0.502 |
| Bonferroni | 0.6 | 75 | 991 | 2409 | 2 | 1998 | 1.184 | 0.002 | 0.291 |
| Raw | 0.6 | 75 | 2154 | 1246 | 86 | 1914 | 1.160 | 0.038 | 0.634 |
| Bayesian | 0.6 | 100 | 2340 | 1060 | 62 | 1938 | 0.990 | 0.026 | 0.688 |
| B-H | 0.6 | 100 | 2171 | 1229 | 33 | 1967 | 1.156 | 0.015 | 0.639 |
| Bonferroni | 0.6 | 100 | 1468 | 1932 | 1 | 1999 | 1.161 | 0.001 | 0.432 |
| Raw | 0.6 | 100 | 2477 | 923 | 93 | 1907 | 1.159 | 0.036 | 0.729 |
| Bayesian | 0.6 | 150 | 2684 | 716 | 67 | 1933 | 1.001 | 0.024 | 0.789 |
| B-H | 0.6 | 150 | 2587 | 813 | 39 | 1961 | 1.106 | 0.015 | 0.761 |
| Bonferroni | 0.6 | 150 | 2049 | 1351 | 2 | 1998 | 1.112 | 0.001 | 0.603 |
| Raw | 0.6 | 150 | 2744 | 656 | 89 | 1911 | 1.106 | 0.031 | 0.807 |
| Bayesian | 0.7 | 25 | 797 | 3003 | 36 | 1564 | 0.928 | 0.043 | 0.210 |
| B-H | 0.7 | 25 | 326 | 3474 | 12 | 1588 | 0.688 | 0.036 | 0.086 |
| Bonferroni | 0.7 | 25 | 62 | 3738 | 0 | 1600 | 0.557 | 0.000 | 0.016 |
| Raw | 0.7 | 25 | 1122 | 2678 | 82 | 1518 | 1.844 | 0.068 | 0.295 |
| Bayesian | 0.7 | 50 | 1653 | 2147 | 60 | 1540 | 1.016 | 0.035 | 0.435 |
| B-H | 0.7 | 50 | 1280 | 2520 | 32 | 1568 | 1.196 | 0.024 | 0.337 |
| Bonferroni | 0.7 | 50 | 564 | 3236 | 5 | 1595 | 1.207 | 0.009 | 0.148 |
| Raw | 0.7 | 50 | 1986 | 1814 | 104 | 1496 | 1.385 | 0.050 | 0.523 |
| Bayesian | 0.7 | 75 | 2181 | 1619 | 48 | 1552 | 0.975 | 0.022 | 0.574 |
| B-H | 0.7 | 75 | 1937 | 1863 | 28 | 1572 | 1.182 | 0.014 | 0.510 |
| Bonferroni | 0.7 | 75 | 1112 | 2688 | 3 | 1597 | 1.208 | 0.003 | 0.293 |
| Raw | 0.7 | 75 | 2382 | 1418 | 70 | 1530 | 1.196 | 0.029 | 0.627 |
| Bayesian | 0.7 | 100 | 2581 | 1219 | 58 | 1542 | 1.008 | 0.022 | 0.679 |
| B-H | 0.7 | 100 | 2420 | 1380 | 37 | 1563 | 1.163 | 0.015 | 0.637 |
| Bonferroni | 0.7 | 100 | 1625 | 2175 | 6 | 1594 | 1.162 | 0.004 | 0.428 |
| Raw | 0.7 | 100 | 2701 | 1099 | 75 | 1525 | 1.158 | 0.027 | 0.711 |
| Bayesian | 0.7 | 150 | 2987 | 813 | 48 | 1552 | 1.006 | 0.016 | 0.786 |
| B-H | 0.7 | 150 | 2899 | 901 | 31 | 1569 | 1.101 | 0.011 | 0.763 |
| Bonferroni | 0.7 | 150 | 2222 | 1578 | 3 | 1597 | 1.106 | 0.001 | 0.585 |
| Raw | 0.7 | 150 | 3037 | 763 | 57 | 1543 | 1.105 | 0.018 | 0.799 |
| Bayesian | 0.8 | 25 | 895 | 3505 | 26 | 974 | 1.000 | 0.028 | 0.203 |
| B-H | 0.8 | 25 | 319 | 4081 | 3 | 997 | 0.744 | 0.009 | 0.073 |
| Bonferroni | 0.8 | 25 | 75 | 4325 | 0 | 1000 | 0.698 | 0.000 | 0.017 |
| Raw | 0.8 | 25 | 1270 | 3130 | 40 | 960 | 1.840 | 0.031 | 0.289 |
| Bayesian | 0.8 | 50 | 1986 | 2414 | 29 | 971 | 1.057 | 0.014 | 0.451 |
| B-H | 0.8 | 50 | 1588 | 2812 | 11 | 989 | 1.247 | 0.007 | 0.361 |
| Bonferroni | 0.8 | 50 | 685 | 3715 | 1 | 999 | 1.256 | 0.001 | 0.156 |
| Raw | 0.8 | 50 | 2302 | 2098 | 48 | 952 | 1.366 | 0.020 | 0.523 |
| Bayesian | 0.8 | 75 | 2572 | 1828 | 36 | 964 | 1.122 | 0.014 | 0.585 |
| B-H | 0.8 | 75 | 2333 | 2067 | 22 | 978 | 1.254 | 0.009 | 0.530 |
| Bonferroni | 0.8 | 75 | 1309 | 3091 | 2 | 998 | 1.272 | 0.002 | 0.298 |
| Raw | 0.8 | 75 | 2745 | 1655 | 48 | 952 | 1.311 | 0.017 | 0.624 |
| Bayesian | 0.8 | 100 | 2810 | 1590 | 27 | 973 | 1.016 | 0.010 | 0.639 |
| B-H | 0.8 | 100 | 2633 | 1767 | 19 | 981 | 1.154 | 0.007 | 0.598 |
| Bonferroni | 0.8 | 100 | 1652 | 2748 | 0 | 1000 | 1.158 | 0.000 | 0.375 |
| Raw | 0.8 | 100 | 2947 | 1453 | 43 | 957 | 1.153 | 0.014 | 0.670 |
| Bayesian | 0.8 | 150 | 3327 | 1073 | 46 | 954 | 1.025 | 0.014 | 0.756 |
| B-H | 0.8 | 150 | 3217 | 1183 | 38 | 962 | 1.119 | 0.012 | 0.731 |
| Bonferroni | 0.8 | 150 | 2394 | 2006 | 2 | 998 | 1.114 | 0.001 | 0.544 |
| Raw | 0.8 | 150 | 3378 | 1022 | 53 | 947 | 1.116 | 0.015 | 0.768 |
