# Supplementary material for: A Multilevel Bayesian Approach to Improve Effect Size Estimation in Regression Modeling of Metabolomics Data Utilizing Imputation with Uncertainty
Source: Metabolites. 2020 Aug 6;10(8):319. doi: 10.3390/metabo10080319 (PMC7465156; doi:10.3390/metabo10080319)
Supplement: Supplementary file 1 [file metabolites-10-00319-s001.zip › Table S2.docx]

**Table S2:** Model performance across varying simulation parameters in the presence of missing data.

| ***Model***  ***Method***^ | ***Average missing rate*** | ***Fraction of Significant Metabolites*** | ***Sample Size***  ***per Group*** | ***True Positives*** | ***False Negatives*** | ***False***  ***Positives*** | ***True***  ***Negatives*** | ***Average Exaggeration Ratio*** | ***FDR^*^*** | ***Power***^†^ |
| --- | --- | --- | --- | --- | --- | --- | --- | --- | --- | --- |
| Bayesian | 0.01 | 0.4 | 100 | 1538 | 662 | 109 | 3091 | 0.917 | 0.066 | 0.699 |
| B-H | 0.01 | 0.4 | 100 | 1407 | 793 | 60 | 3140 | 1.152 | 0.041 | 0.64 |
| Bonferroni | 0.01 | 0.4 | 100 | 1060 | 1140 | 4 | 3196 | 1.175 | 0.004 | 0.482 |
| Raw | 0.01 | 0.4 | 100 | 1679 | 521 | 175 | 3025 | 1.144 | 0.094 | 0.763 |
| Bayesian | 0.05 | 0.4 | 100 | 1548 | 652 | 110 | 3090 | 0.936 | 0.066 | 0.704 |
| B-H | 0.05 | 0.4 | 100 | 1443 | 757 | 69 | 3131 | 1.212 | 0.046 | 0.656 |
| Bonferroni | 0.05 | 0.4 | 100 | 1093 | 1107 | 6 | 3194 | 1.233 | 0.005 | 0.497 |
| Raw | 0.05 | 0.4 | 100 | 1707 | 493 | 183 | 3017 | 1.204 | 0.097 | 0.776 |
| Bayesian | 0.1 | 0.4 | 100 | 1552 | 637 | 128 | 3056 | 0.97 | 0.076 | 0.709 |
| B-H | 0.1 | 0.4 | 100 | 1473 | 716 | 91 | 3093 | 1.302 | 0.058 | 0.673 |
| Bonferroni | 0.1 | 0.4 | 100 | 1116 | 1073 | 10 | 3174 | 1.318 | 0.009 | 0.51 |
| Raw | 0.1 | 0.4 | 100 | 1735 | 454 | 209 | 2975 | 1.295 | 0.108 | 0.793 |
| Bayesian | 0.15 | 0.4 | 100 | 1574 | 626 | 129 | 3071 | 0.991 | 0.076 | 0.715 |
| B-H | 0.15 | 0.4 | 100 | 1504 | 696 | 94 | 3106 | 1.393 | 0.059 | 0.684 |
| Bonferroni | 0.15 | 0.4 | 100 | 1153 | 1047 | 11 | 3189 | 1.406 | 0.009 | 0.524 |
| Raw | 0.15 | 0.4 | 100 | 1749 | 451 | 212 | 2988 | 1.384 | 0.108 | 0.795 |
| Bayesian | 0.2 | 0.4 | 100 | 1579 | 610 | 147 | 3037 | 1.016 | 0.085 | 0.721 |
| B-H | 0.2 | 0.4 | 100 | 1519 | 670 | 111 | 3073 | 1.472 | 0.068 | 0.694 |
| Bonferroni | 0.2 | 0.4 | 100 | 1159 | 1030 | 16 | 3168 | 1.486 | 0.014 | 0.529 |
| Raw | 0.2 | 0.4 | 100 | 1752 | 437 | 238 | 2946 | 1.465 | 0.12 | 0.8 |
| Bayesian | 0.25 | 0.4 | 100 | 1602 | 598 | 167 | 3033 | 1.046 | 0.094 | 0.728 |
| B-H | 0.25 | 0.4 | 100 | 1543 | 657 | 138 | 3062 | 1.544 | 0.082 | 0.701 |
| Bonferroni | 0.25 | 0.4 | 100 | 1173 | 1027 | 21 | 3179 | 1.556 | 0.018 | 0.533 |
| Raw | 0.25 | 0.4 | 100 | 1766 | 434 | 263 | 2937 | 1.548 | 0.13 | 0.803 |
| Bayesian | 0.3 | 0.4 | 100 | 1600 | 589 | 180 | 3004 | 1.071 | 0.101 | 0.731 |
| B-H | 0.3 | 0.4 | 100 | 1554 | 635 | 154 | 3030 | 1.631 | 0.09 | 0.71 |
| Bonferroni | 0.3 | 0.4 | 100 | 1176 | 1013 | 26 | 3158 | 1.635 | 0.022 | 0.537 |
| Raw | 0.3 | 0.4 | 100 | 1765 | 424 | 311 | 2873 | 1.633 | 0.15 | 0.806 |
